# The REACT MCI study: a randomized controlled clinical trial on the effect of repeated advanced cognitive training in mild cognitive impairment

**DOI:** 10.1186/s13063-026-09711-6

**Published:** 2026-05-07

**Authors:** S. S. Hernes, P. R. Nordnes, H. R. Hol, G. Ringstad, A. B. Knapskog, P. Emhjellen, G. Løhaugen, M. Emini, T. H. Edwin

**Affiliations:** 1 Department of Geriatric and Internal Medicine, Sorlandet Hospital Arendal HF, Arendal, Norway; 2 Department of Clinical Sciences, Faculty of Medicine, University of Bergen, Bergen, Norway; 3 KG Jebsen Centre for Brain fluid research, Oslo, Norway; 4 Department of Radiology, Oslo University Hospital, Oslo, Norway; 5 University of Oslo, Oslo, Norway; 6 Department of Geriatrics, Oslo University Hospital, Oslo, Norway; 7 Department of Pediatrics, Sorlandet Hospital Arendal, Arendal, Norway; 8 Norwegian Health Association, Oslo, Norway

**Keywords:** Mild cognitive impairment, Working memory training, Quality of life, Cost-effectiveness, Proxy

## Abstract

**Background:**

Mild cognitive impairment (MCI) and its course have multiple determining factors with both detrimental and compensatory processes in the brain. Computerized working memory training can improve cognitive function and individuals with MCI are potential targets. Since more than 1/3 of these individuals develop dementia within 5 years, any intervention postponing the progression will be immensely important. We aim to investigate the efficiency and cost-effectiveness of working memory training in MCI.

**Methods:**

In this blinded randomized controlled trial, 213 participants will be allocated to three groups: (1) one 5-week period of working memory training, (2) two 5-week periods of working memory training, or (3) the active control group. The participants are followed for a period of 48 months (3, 6, 12, 24, and 48 months). Furthermore, we investigate microstructural effects of working memory training on magnetic resonance imaging and seek to identify high responders to training by assessing the association with function in the glymphatic system and genetic variations. Finally, the impact of working memory training on quality of life and relatives’ stress, and whether it is a cost-effective approach in MCI, will be assessed.

**Discussion:**

Effective therapy for MCI is lacking. Finding an intervention postponing the progression of MCI is of great importance for patients as well as for the global economy and health care.

**Trial registration:**

ClinicalTrials.gov NCT04792528. Registered on 02.23.2021.

## Administrative information

Note: the numbers in curly brackets in this protocol refer to SPIRIT checklist item numbers. The order of the items has been modified to group similar items.

**Table:** 

Title {1}	The REACT MCI study: a randomized controlled clinical trial on the effect of repeated advanced cognitive training in mild cognitive impairment
Trial registration {2a and 2b}	Clinical trials identifier: NCT04792528. Registered 02.23.2021. Prospectively registered
Protocol version {3}	Protocol version 3.02 date 03.04.2024
Funding {4}	The project is funded by Klinisk behandlingsforskning (klinbeforsk) for the period of 2021-2026. Project nr 2019208, South Eastern Regional Health Authority, project nr 2023085. Through the research department at Sorlandet Hospital trust a private donor has provided additional funding for the study.
Author details {5a}	Susanne Hernes, MD, Phd 1. Department of geriatric and internal medicine, Sorlandet hospital Arendal HF 2. Department of clinical sciences, Faculty of medicine, University of Bergen Trine Holt Edwin 1. Department of Geriatric Medicine, Oslo University Hospital
Name and contact information for the trial sponsor {5b}	KLINBEFORSK klinbeforsk@helse-sorost.no. South Eastern health care authority forskningsmidler@helsesor-ost.no
Role of sponsor {5c}	The sponsor played no part in planning the study design. The sponsor will not play any part in the collection, management, analysis, and interpretation of data; writing of the report; and the decision to submit the report for publication.

## Introduction

### Background and rationale {6a}

The cognitive and functional impairment in dementia develops slowly and the neuropathological process often begins decades before the clinical signs emerge [1]. This has led to the recognition of a prodromal period of mild cognitive impairment (MCI) between normal cognition and dementia [2]. Individuals with MCI have an increased risk of developing dementia as compared to the population as a whole [3]; any intervention postponing the progression will be immensely important. For the US, a mere one-year delay in dementia onset leads to an estimated 219 billion USD in reduced costs by 2050 [4]. Currently, there is no curative treatment for MCI.

Upon repeated cognitive stimulation, the mammal brain can build and improve myelination of neurons and increase synaptic density [5]. This neuroplasticity gives rise to a new intervention avenue in memory disorders: training core brain pathways to increase cognitive function. The Scaffolding Theory of Aging and Cognition (STAC) proposes that cognitive training can promote “compensatory scaffolding” in the brain by creating additional neural connections [6]. The Cognitive Reserve Theory supports this idea by highlighting the dynamic relationship between cognitive stimulation and a decreased risk of dementia [7, 8]. Studies suggest that computerized cognitive training can improve neuropsychological test performance in unimpaired older adults [9, 10], and in some, the improvement is maintained for up to 10 years [11]. Individuals with MCI are ideal targets for computerized cognitive training as their impairment is at an early stage; they are concerned and, in most cases, able to utilize computerized interventions.

Difficulties with working memory, the ability to select and focus on information for shorter periods of time, are common in MCI [12], and impact daily cognitive functioning [13]. Working memory relies on an extensive neural network that connects the frontal and parietal lobes with posterior sensory cortices [14]. Given its role in core brain pathways, working memory is a promising target for restorative interventions.

Identification of individuals with genetic profiles that are more likely to experience effects of computerized working memory training is central to providing tailored care for individuals with MCI. Dopamine plays a key role in memory formation by increasing the working memory function and modulating plasticity within the ventral striatum [15]. The Lim homeobox transcription factor 1 alpha (*LMX1a*) gene is closely associated with the development and maintenance of dopaminergic neurons [16]. In individuals with HIV-associated neurocognitive disorder, carriers of the *LMX1a-AA* allele displayed greater cognitive training gain [17], whereas in cognitively unimpaired individuals conflicting results exist [18, 19]. The *COMT* gene encoding for Catechol-O-Methyl transferase is another potential marker for improved cognitive training gain as the Val158Met variant impacts intracerebral dopamine breakdown [18]. Another potential indicator of the efficacy of cognitive training is the Apolipoprotein E (*APOE*) gene, whose Ɛ4 allele is a risk factor for AD [20]. According to the antagonistic pleiotropic hypothesis the *APOE Ɛ4* affects cognition beneficially in early and midlife while the disadvantageous effect associated with increased risk of AD appears in late life [21]. Engaging in cognitive activities throughout life may diminish the negative effect of *APOE Ɛ4* on later-life cognition [22]. This leaves a theoretical window for influencing cognitive function before the AD pathological process progresses. Moreover, a novel genetic marker, the aquaporin 4 (AQP4) gene, is of interest as it encodes cerebral water channels that are involved in synaptic plasticity and memory formation [23]. A brain-wide, perivascular pathway for clearance of toxic brain solutes was discovered in 2012 [24]; offering evidence for a system-dependent effect of AQP4 at astrocytic end feet denoted the glia-lymphatic, or glymphatic system. These lymphatic vessels are deemed crucial to glymphatic clearance function, and their impairment is linked to deteriorated cognitive function [25–27].

In this study, we aim to investigate the effect of computerized working memory training in individuals with MCI. We developed four work packages to (1) examine the effects of working memory training on cognitive test performance, (2) identify high responders to working memory training, (3) assess its impact on quality of life, and (4) evaluate its socioeconomic implications.

Five Norwegian memory clinics with radiology departments in four health care regions have teamed with major US cognitive research groups to perform this investigation, which challenges current treatment of MCI.

### Objectives {7}

The primary objective of the present study is to evaluate the overriding hypothesis that working memory training is an efficient and cost-effective intervention in MCI as compared to an active control group. To test this hypothesis, we have formulated four aims, giving rise to four work packages.

Work package 1: We aim to investigate if a single period of working memory training is sufficient to achieve effects on cognitive function after 6 months measured by spatial span backwards. To assess potential transfer effects, we will further examine whether working memory training impacts memory, attention, visuospatial abilities, language, executive function, or behavior (neuropsychiatric symptoms) are impacted by training. Moreover, we will investigate a possible dose-dependent effect of working memory training by comparing the group receiving two periods of working memory training with those receiving one period. Microstructural effects of working memory training on cerebral white matter after 6 months measured by quantitative magnetic resonance imaging (MRI) will be explored. We will perform subgroup analyses on difference in training effects between amnestic MCI and non-amnestic MCI, according to different etiologies of MCI, and according to the presence of amyloid, tau or neurodegeneration.

Work package 2: The aim is to investigate possible characteristics of high responders of working memory training regarding demographic and clinical factors and allelic variations in LIM homeobox transcription factor 1 alpha(*LMX1a*), Catechol-O-methyltransferase (*COMT*), Apolipoprotein epsilon (*APOE*), Aquaporine 4 (*AQP4*), Aquaporine 4 anchoring protein (*AQO4*). The influence of the glymphatic system will be examined by testing how craniospinal clearance rate is associated with working memory function and training effect. Lastly, in work package 2, we will investigate working memory training effects on gray matter evaluated by quantitative MRI techniques.

Work package 3: The aim is to investigate if working memory training impacts quality of life (for both participant and proxy) and relative’s stress.

Work package 4: The aim is to evaluate whether working memory training is cost-effective in MCI. The quality adjusted life years (QALY) associated costs and the use of health care resources in the patient and relative population will be investigated in the MCI study population as a whole and by training group.

## Methods: participants, interventions and outcomes

### Study setting {9} recruitment {15} and eligibility criteria {10}

The trial will include individuals diagnosed with MCI in Norway, recruited from or through the following Norwegian memory clinics; Kløveråsen Memory Clinic and Old-Age Psychiatry Unit (Bodø), St. Olav’s University Hospital Geriatric Outpatient Department (Trondheim), Olaviken Old-Age Psychiatry Unit (Bergen), Oslo University Hospital Memory Clinic (Oslo) and Sørlandet Hospital Arendal Clinic for Ageing and Memory (Arendal). The clinical team at the study centers identifies eligible individuals. A research nurse will then contact the individuals and provide detailed information sheets by post. The research nurse will conduct a follow-up telephone call to establish whether to arrange an appointment for a baseline assessment. At baseline, a researcher confirms eligibility and receives consent of participation. We will register the number of individuals not meeting the inclusion criteria or declining participation.

Eligible for inclusion are individuals meeting the diagnostic criteria of MCI based on the Mayo/Winblad 2004 criteria [28]:

1. Not cognitively normal but does not meet the Diagnostic and Statistical Manual of Mental Disorders (DSM) IV or International Classification of Diseases and Related Health Problems, 10th edition (ICD-10) criteria for dementia.
2. The presence of cognitive decline. The cognitive decline should be self-reported or described by an informant, or there should be evidence of cognitive decline over time on objective tasks.
3. Intact activities of daily living or minimal impairment in complex instrumental activities.

There is no clear consensus regarding subnormal performance on neuropsychological tests indicating MCI [29–31]. Yet a score cut-off 1.5 standard deviations (SD) below the mean compared to norms in standardized cognitive tests are commonly applied [32]. Therefore, to ensure agreement, we require age- and education-adjusted scores at least 1.5 SD below the mean on at least one test in one or more cognitive domains to meet the criteria of cognitive decline in MCI.

Furthermore, the participants must have access and be able to use a tablet or computer and be fluent in Norwegian. Cerebrospinal fluid (CSF) biomarkers or an amyloid positron emission tomography (PET) scan, obtained as part of the clinical examination, are recommended but not required. The use of cholinesterase inhibitors is permitted if the participant has been in a stable dose for at least 3 months.

Exclusion criteria:

- Having a major psychiatric illness or current substance abuse
- Having a major somatic illness with expected survival <24 months
- Experiencing non-compensated hearing impairment or visual impairment
- Cerebral stroke within the last 12 months

Additional exclusion criteria for the glymphatic sub-study.

- Known allergy against contrast solutions or other serious allergies
- The use of anticoagulant medication or the use of two or more antiplatelet medication
- Increased bleeding tendency (International normalized ratio > 1,5 and/or platelet level < 100 × 10E9/L)
- Renal failure or an estimated glomerular filtration rate (eGFR) < 30 mL/min/1.73 m^2^. For individuals > 70 years or those with medications affecting kidney function, eGFR must be measured within the last week
- The presence of infection or a tattoo at the site of insertion
- Being younger than 18 or older than 80
- Pregnant or lactating women

### Who will take informed consent? {26a}

Eligible individuals receive oral and written study information by study personnel at each site. The study personnel obtain informed consent from the participants and their relatives.

### Additional consent provisions for collection and use of participant data and biological specimens {26b}

Consent includes the permission to collect whole blood, saliva for DNA extraction and for linkage to The Norwegian Registry of Persons Assessed for Cognitive Symptoms, the Norwegian Registry for Primary Health Care and the Norwegian patient registry. The additional consent for the glymphatic sub-study includes permission to collect plasma after spinal contrast injection.

## Intervention

### Explanation for the choice of comparators {6b}

The trial includes two intervention groups receiving different doses of computerized adaptive working memory training (single training period and double training period) and one active control group receiving computer-based solitaire. Based on previous research [33], an active control group was chosen rather than a passive control group to better control for non-specific effects related to study participation and repeated computer-based activity.

Upon repeated cognitive stimulation, the mammal brain can build and improve the myelination of neurons and increase synaptic density [5]. This neuroplasticity gives rise to a new intervention avenue in memory disorders: training core brain pathways to increase cognitive function. Working memory is a core function that involves structures and processes linked to the ability to retain information, while actively using and responding to the information to guide behavior [34]. Working memory is thus related to reasoning, learning and comprehension [34]. In order for an intervention to be used as a delaying treatment in MCI, the training needs to (1) target a core function such as working memory and (2) be effective also for non-trained tasks (transfer effects) by improving cognitive functions requiring the same intracerebral processes and neural connections as the trained tasks [35]. Working memory training thus holds promise as a potential intervention, targeting core cognitive functions and promoting transfer effects that may support broader cognitive resilience in individuals with MCI.

### Trial design {8} and intervention description {11a}

#### Working memory training

This is a Norwegian multi-center, blinded three-arm, parallel-group randomized controlled trial (RCT) with block randomization and an allocation ratio of 1:1:1. The trial is designed within a superiority framework to evaluate whether adaptive working memory training improves outcomes compared with an active control condition, and to explore whether repeated training provides additional benefit (dose–response effect). The COGMED® program (Neural Assembly Int AB, Sweden) is a computerized adaptive program designed through repetitive tasks to improve working memory. One training period lasts about 5 weeks and consists of 40 min of daily adaptive training for 4–5 days a week, resulting in 20–25 training sessions. Individuals log on to a portal to train in the privacy of their homes, thus enabling logging of training time. The participants are randomized at study inclusion to one of three groups: (1) 5 weeks of adaptive working memory training (20–25 sessions), (2) 5 weeks of adaptive working memory training (20–25 sessions), repeated twice 1–2 months apart, or (3) 5 weeks (20–25 sessions) of Klondike solitaire on an online portal (active control group). These individuals self-report training frequency and times through a written log. All groups receive individual weekly coaching as per requisite from the COGMED® program. The use of an active control groups is based on the international consensus to improve quality of computerized cognitive intervention studies, as described by Simons et al. [33].

#### Assessment of clearance from cerebrospinal fluid to blood

A subset of participants from Oslo University Hospital will be included in a sub-study where craniospinal clearance to blood as a surrogate marker of glymphatic function is measured at baseline and after 24 months. The procedure includes a 3 ml injection of Iodixanol 270 mg I/ml (Visipaqueᴿ, GE Healthcare) in the spinal canal by lumbar puncture at the level of L4/L5 using a standardized needle of 22G. We collect plasma 2 and 4 h (±1 h) after intrathecal contrast injection. One aliquot will be used for analysis of Visipaque concentration; the remainder will be stored at −80 °C at Oslo University Hospital pending further analyses.

#### Assessments

**Fig. 1:**
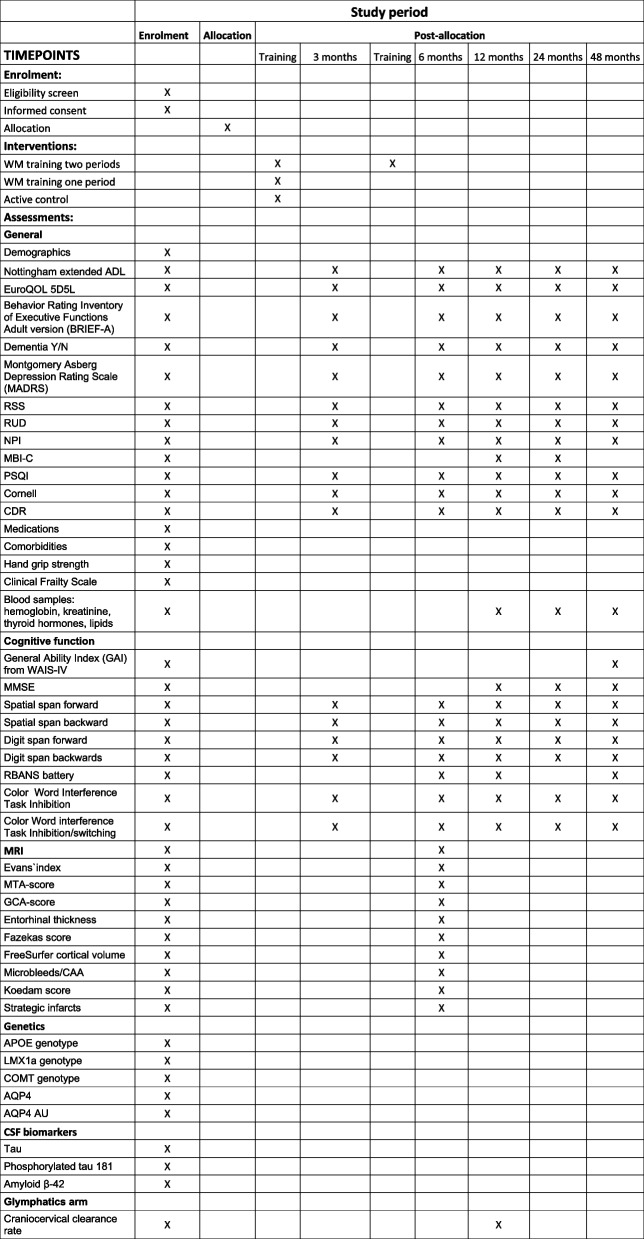
SPIRIT figure

Baseline assessments are completed prior to randomization; thereafter, the participant will be followed at 3, 6, 12, 24, and 48 months (Fig. 1).

At baseline, the research protocol includes information on sex, age, years of education, medication use, family history of dementia (first-degree relatives) within the following disease categories: cerebral stroke, myocardial infarction, hypertension, and thyroid dysfunction. Blood pressure, body mass index and handgrip strength at baseline will be obtained and the Clinical Frailty Scale [36] will be scored.

The participants’ cognitive and functional abilities, neuropsychiatric symptoms and quality of life will be assessed at baseline, 3, 6, 12, 24 and 48 months by commonly applied tests, questionnaires and interviews (Fig. 1).

The General Ability Index (Wechsler Adult Intelligence Scale, 4ed) [37] will be scored at baseline as a measure of intellectual functioning to detect low-functioning cognitively normal persons or high-functioning persons with dementia misclassified as having MCI.

The participants’ cognitive abilities within the domains: memory, working memory, attention, visuospatial abilities, language and executive function will be assessed by standardized neuropsychological tests. On repeated tests, alternative test versions will be used, when available, to correct for learning effects. The selected neuropsychological tests and time points of assessments are listed in Table 1.

**Table 1:** Assessment of intellectual functioning and cognitive domains

Domain	Description	Time points
Intellectual functioning*	General Ability Index (GAI)	Baseline
Global screening	MMSE	Baseline, 12, 24, and 48 months
Working memory**	Spatial span forward, spatial span backward, digit span forward, and digit span backwards	Baseline, 3, 6, 12, 24, and 48 months
Immediate learning***	List learning and story memory	Baseline, 6, 24, and 48 months
Visuospatial***	Figure copy and line orientation	Baseline, 6, 24, and 48 months
Language***	Picture naming and Semantic fluency	Baseline, 6, 24, and 48 months
Attention***	Digit span and coding	Baseline, 6, 24, and 48 months
Delayed memory***	List recall, list recognition, story memory and figure recall	Baseline, 6, 24, and 48 months
Executive function****	Color Word Interference Task Inhibition and Color Word interference Task Inhibition/switching	Baseline, 3, 6, 12, 24, and 48 months

*Abbreviations*: *MMSE *Mini-Mental State Examination

*GAI from the Wechsler Adult Intelligence Scale 4ed

**From the Wechsler Memory Scale 3.ed

***From the Repeatable Battery for the Assessment of Neuropsychological Status (RBANS) test battery

****From the Delis–Kaplan test battery

### Plans for collection, laboratory evaluation, and storage of biological specimens for genetic or molecular analysis in this trial/future use {33}

#### Magnetic resonance imaging (MRI)

MRI images will be assessed and scored using Evans’ index, the medial temporal lobe atrophy score (Scheltens’ score), the global cortical atrophy scale (GCA score), the white matter lesions score (Fazekas’ score), the parietal atrophy score (Koedam score), evaluation of microbleeds (MARS score) and identification of any strategic infarcts affecting areas that are critical for normal cognitive functioning of the brain (presence). 

Additionally, the study will quantify grey matter properties by assessing thickness, area, and local gyrification index on T1 and T2 images using Freesurfer version 7.0 or higher, as well as white matter microstructure changes with diffusion tensor imaging (DTI) by the different diffusivity parameters both at baseline and longitudinally.

Quantitative susceptibility mapping (QSM) is a novel technique for stratifying cerebral microbleeds and amyloid beta plaques by measuring the redox-iron accumulated near the amyloid plaques and in the iron-dependent cell death pathway (ferroptosis) associated with Alzheimer’s disease [38–40]. QSM values are increased in MCI patients compared to cognitively normal older adults. QSM has been reported to better differentiate individuals with amnestic MCI from cognitively normal individuals compared to measurements of grey matter volumes [41]. The association between QSM values and cognitive training effects will be assessed.

Arterial spin-labelling MRI is a noninvasive technique that uses magnetically labeled arterial blood water as an endogenous contrast medium for the evaluation of cerebral blood flow. The technique gathers indirect data about glucose metabolism in the brain, by correlating the blood flow to regions. This technique has been shown to be associated with cognitive training effects and may have implications for working memory trainability.

Previous studies have found correlations between resting cerebral blood flow, as measured by ASL, and cognitive training outcomes in patients with mild cognitive impairment (MCI). We will investigate the potential of ASL to predict trainability outcomes in MCI patients.

 Magnetic resonance elastography will be used to non-invasively assess tissue microstructural integrity by measuring brain viscoelastic mechanical properties and correlated with glymphatic function.

#### Blood

All participants will provide blood samples with information about levels of hemoglobin, creatinine, thyroid hormones and lipids (total cholesterol, low density lipoprotein and high density lipoprotein) at baseline, 12 and 24 months. Plasma, for the analysis of phosphorylated tau 217, will be harvested from participants who did not undergo a lumbar puncture. In the glymphatic sub-study plasma will be collected, see intervention description {11a}.

#### Cerebrospinal fluid (CSF)

At baseline, the participants will be categorized according to the AT(N) research classification system [38] as normal AD biomarkers (A-T-[N]-), Alzheimer’s continuum (A+T-[N]-, A+T+[N]-, A+T+[N]+, and A+T-[N]+) or non-Alzheimer’s pathologic change (A-T+[N]-, A-T-[N]+and A-T+[N]+). The presence of amyloid β (Aβ) deposition (A) will be determined by the level of Aβ in the CSF, by phosphorylated-tau in plasma, or by an amyloid PET scan. Pathologic tau (T) will be determined by the CSF level of phosphorylated-tau181 (p-tau). The presence of neurodegeneration (N) will be detected by structural MRI, by a glucose PET scan, or by the CSF level of total tau (t-tau).

#### Saliva

Saliva will be collected by the use of OG-600 Oragene DNA (DNAGenotec, Canada) at baseline for genotype analyses of APOE, LMX1a, COMT, AQP4 and AQP4 AU alleles.

The saliva for genetic analyses will be stored at room temperature within a secure locker and will be destroyed after analysis.

### Strategies to improve adherence to interventions {11c}

All participants will be coached during the training period, regardless of study group allocation. Coaching will consist of a weekly phone conversation by a member of the study group to offer motivation and advice during the training phase of the intervention, a total of 6 calls per participant. In study arm 1 (repeated COGMED training), the participants will receive an additional 6 calls during training period two. The coaches are allocated to the participants by chance. A coaching phone call is estimated to last approximately 30 min.

### Criteria for discontinuing or modifying allocated interventions {11b}

The intervention will be discontinued if the participant, for any given reason, does not complete the planned 20–25 sessions. In such an event, the participant will be invited to remain in the study and continue the follow-up visits to harvest the non-training data.

### Relevant concomitant care permitted or prohibited during the trial {11d} and provisions for post-trial care {30}

All participants will receive usual care at the clinics throughout the trial. Standard care is provided within the Norwegian health care system.

## Outcomes {12}

### Primary outcome

The primary endpoint is the change in working memory function after 6 months measured by Spatial Span Backwards (from the Wechsler Memory Scale, third edition (WMS-III)) [39, 40].

To investigate immediate and long-term effects, the intervention groups will also be compared to the active control at 3, 12, 24, and 48 months. We will investigate a possible dose-dependent effect by comparing working memory performance, measured by spatial span backwards at 6, 12, 24, and 48 months, between the group receiving two periods of working memory training and the group receiving one period.

### Secondary outcomes

#### Clinical outcomes

Secondary cognitive endpoints are scores on tests within the working memory domain at 3, 6, 12, 24, and 48 months post intervention assessments. Moreover, secondary endpoints are the RBANS composite score, scores on the executive function domain, scores on the Mini-Mental State Examination (MMSE), the Clinical Dementia Rating scale (total and sum of boxes), and conversion to dementia.

We consider the participants as having dementia when they fulfill the National Institute on Aging and Alzheimer’s Association (NIA-AA) 2011 criteria for all-cause dementia [41]:

Cognitive or behavioral symptoms that:

1. Hinder the ability to function independently in activities of daily living or work
2. Constitute decrease in functioning and performing from previous levels
3. Could not be explained by delirium or a major psychiatric disorder
4. Cognitive impairment detected through history taking and cognitive testing
5. Involves at least two of the following domains:
  Immediate learning and delayed memory (list learning, story memory, list recall, list recognition, story memory and figure recall index score from RBANS test battery)
  Reasoning, judgement and handling of complex tasks (Clinical Dementia Rating Scale, CDR).
  Visuospatial abilities (figure copy and line orientation index score from the RBANS test battery)
  Language (picture naming and semantic fluency index score from RBANS test battery)
  Behavioral and psychological symptoms (Neuropsychiatric Inventory Questionnaire (NPI-Q), and Mild Behavioral Impairment Checklist (MBI-C))

We require age- and education-adjusted scores at least 1.5 SD below the mean to consider a cognitive domain as affected. Affected behavior requires a minimum of one domain with the severity score 2 (moderate) or 3 (severe) or a NPI-Q sum score ≥ 3. To ensure consistency, dementia diagnoses will be discussed in consensus meetings involving experienced geriatricians and neuropsychologists.

#### MRI

The effects of working-memory training on cerebral white-matter microstructure will be measured by quantitative magnetic resonance imaging (MRI) (Work package 1).

Investigate WM-training effects on gray matter evaluated by quantitative MRI techniques (Work package 2).

Assessment of ASL as a predictor of trainability in MCI patients (Work package 2).

#### High responders/subgroups (work package 2)

We will perform subgroup analyses on differences in training effects to identify high responders according to:

o Amnestic MCI and non-amnestic MCI
  Different etiologies of MCI
  The presence of amyloid, tau or neurodegeneration
  Allelic variations in APOE, LMX1a, COMT, AQP4, and AQP4 AU alleles
  Cerebral white and grey matter evaluated by quantitative MRI techniques
  Association with QSM values attained by MRI
  Frailty
  Sleep quality
  Neuropsychiatric symptoms

Furthermore, in the glymphatic sub-study we will assess the influence of the glymphatic system by testing how the craniospinal clearance rate is associated with working memory function and training effect. Parameters of craniospinal clearance function, including absorption half-life (T1/2, abs), time to maximum concentration (Tmax), and lag-time of absorption to blood (Tlag), were used as surrogate markers of brain glymphatic clearance function, since previous studies have shown a high association between CSF clearance and brain clearance. The clearance parameters are based on previous pharmacokinetic modeling studies.

#### Quality of life and relatives stress (work package 3)

Participant and relative quality of life (QoL), as well as relative stress, will be measured at baseline and at 3, 6, 12, 24, and 48 months post-intervention.

The next-of-kin completes the Relative stress scale (RSS), a self-administered questionnaire assessing various aspects of proxy distress divided into the subscales “emotional distress,” “social distress,” and “negative emotions.”

QoL of participants and their next-of-kin will be assessed, with EQ-5D-5L, at baseline and at 3, 6, 12, 24, and 48 months post-intervention. Additionally, participants and their next-of-kin will complete the BRIEF-A at the aforementioned intervals. The BRIEF-A assesses executive function and self-regulation in daily life, covering two main indexes (metacognition and behavioral regulation), along with an overall summary score and three validity indicators. The BRIEF-A provides an indirect measure of QoL and relative stress, by assessing how executive function impacts daily life of the participants.

The combination of RSS, EQ-5D-5L, BRIEF-A, in addition to the Neuropsychiatric Inventory Questionnaire ensures solid information on QoL and relative stress.

#### Use of health care resources and health economics (work package 4)

A cost-utility analysis will be performed to evaluate if the working memory training is more cost-effective compared to the active control group in the MCI phase. The generic outcome in the analysis is QALYs derived from the instrument EQ-5D 5L. The secondary outcome is productivity loss of relatives and patients.

The use of QALY as a patient-reported outcome measure in dementia research is debated, as concerns arise regarding patients’ potential unawareness of their impairments or difficulty accurately reporting their status due to memory limitations. The health economic analysis will therefore include the following costs:

1. Intervention-related costs
2. Direct costs/health care costs, mainly related to the use of health care services, including primary, secondary and tertiary care and health services
3. Indirect costs, including work absence for the patients and the relatives of the patients (i.e., productivity loss).

Data will be collected through self-report measures (EQ-5D 5L) and from the Norwegian patient registry. Informal care will be measured by interviews with the family using Resource utilization in dementia [42].

### Participant timeline {13}

See Fig. 1 for the participant’s timeline through the trial.

### Sample size {14}

Sample size and power estimations are based on previously published data [43], assumed effect sizes (Cohen’s *d*) in the study population, and a comparison of two groups with equal sample sizes for simplicity. Stata 15 was used for the estimations, assuming an 80% power (beta = 0.8) and a 5% significance level (alpha = 0.05).

Work package 1: A comparable study reported a mean Spatial Span Backwards score of 5.80 (SD 2.00) at baseline in MCI [43]. A clinically significant effect between groups is defined as a mean difference of 0.5 times the standard deviation, resulting in a required sample size of 64 participants per group. With three groups, this totals 192 participants. Accounting for an anticipated 10% dropout rate, we plan to include a total of 213 (71 in each group).

Work package 2: Genetic variation and craniospinal clearance rate may independently influence or modify the effect of working memory training. The planned sample size is adequate to detect an effect comparable to the expected intervention effect, defined as a mean difference of 0.5 times the standard deviation. We should be able to assess possible effect modification within the intervention groups if it is strong (a Cohen’s *d* population parameter of about 1.0).

Work package 3: A comparable non-dementia study sample reported a mean RSS score of 6.6 (SD 8.8) [44]. Although no minimal clinically important difference has been established, we assume a similar mean difference between groups as estimated for the primary outcome in work package 1 (i.e. Spatial Span backwards score). Thus, the planned total sample size of 213 participants (71 in each group) is considered sufficient.

Work package 4: Assuming an annual dementia transition rate of 9.6% [3], a study population of 213 individuals is projected to yield 39 cases of dementia at the 24-month follow-up and 71 cases at the 48-month follow-up. However, these estimates are affected by uncertainties regarding the transition rate specific to working-memory-training populations and the applicability of international data to a Norwegian cohort.

## Assignment of interventions: allocation

### Sequence generation {16a} and concealment mechanism {16b}

After informed consent is given, participants will be randomized in the Web-CRF system (Norwegian University of Technology and Science) to one of three groups: (1) one 5-week period of working memory training, (2) two 5-week periods of working memory training, or (3) the active control group. The allocation sequence will be generated centrally within the Web-CRF system by a computer-generated randomization procedure, with randomization stratified by study center and restricted by permuted blocks of six to eight to maintain balance between groups within centers. Allocation concealment will be ensured by the Web-CRF system, and allocation is concealed and unavailable to the study team.

After randomization, group allocation is visible only to the Principal Investigator, the study secretary, and the coaches in the Web-CRF portal, who use this information to provide the participant with the correct login details, training materials and support during the training period.

### Implementation {16c}

A unique identifier is automatically generated within the digital Web-CRF system. Participant enrollment is conducted by the research nurse at each site. Following randomization, the Principal Investigator assigns training instructions to participants and communicates these to the coaches. Coaches have restricted access to the patient database and are only aware of the individuals they are currently coaching. No coaches are allowed to coach participants within their own geographic localization.

## Assignment of interventions: blinding

### Who will be blinded {17a}

All study personnel except for the Principal Investigator, coaches and the study secretary will be blinded for group allocation.

### Procedure for unblinding if needed {17b} and interim analyses {21b}

Treatment allocation will remain blinded throughout the trial and will not be disclosed before completion of the 48-month follow-up, except to the Principal Investigator, study secretary and coaches who require allocation information for trial administration and intervention delivery.

To facilitate planned analyses and publications during the study period, PhD candidates and senior researchers may be given access to de-identified datasets after completion of the baseline, 3-month, 6-month, 12-month, 24-month, and 48-month assessments, in which participants are assigned a unique mock study code. This identifier has no traceable link to the participant’s original study ID, and the allocation key will remain inaccessible to blinded study personnel. After all participants have completed the 48-month follow-up, full unblinding of treatment allocation will be performed.

## Data collection and management

### Plans for assessment and collection of outcomes {18a}

Data are collected at baseline and at 3, 6, 12, 24, and 48 months follow-up. The data management system Web-CRF will be used to input, manage and monitor data. Trained study nurses will gather background information, collect data from next-of-kin, and assess neuropsychiatric symptoms and activities of daily living. The EuroQOL 5D5L and BRIEF-A questionnaires will be sent to the participants and their next-of-kin for completion and then returned to the study secretary. The cognitive test battery and the Clinical Frailty Scale will be administered by researchers who are also experienced geriatricians or neuropsychologists. To ensure consistency, these researchers will receive training from a neuropsychologist prior to any testing. Saliva samples for genetic analysis will be collected by researchers and stored at room temperature, as required. Laboratory tests and MRIs are performed by the diagnostic laboratories at the sites. The cost-effectiveness analysis is described under Outcomes {12}.

### Plans to promote participant retention and complete follow-up {18b}

Detailed information about the study set-up and requirements will be sent to the participants before inclusion and the importance of completion will be stressed. Participants can stop the training or withdraw consent at any time without providing a reason to discontinue. In case of training cessation, the participants will be asked, but are not obliged, to complete the follow-up assessments. To promote retention, all participants will be coached during the training period, regardless of study group allocation. The research nurses remind patients of MRI and study-visit appointments, and we aim to coordinate the MRI examinations on the same day as the study visit.

### Data management {19} and plans for auditing trial conduct {23}

The data will be entered to the Web-CRF program on a secure server (Norwegian University of Technology and Science) by site staff. The Principal Investigator monitors and ensure that missing data are addressed as soon as possible. Datasets will be locked for further input after all participants have passed each milestone (3 months, 6 months, 12 months, 24 months, and 48 months). Informed consent, test material and questionnaires will be stored within a secure locker in a locked room. All MRI data acquired will be deidentified and saved in a study folder on a protected national research server.

### Confidentiality {27}

Study personnel and members of all boards are bound by the Norwegian health care confidentiality described in Health Personnel Act §5. Furthermore, no study results can be used or published without the approval of the Principal Investigator. Data is stored using a study identification code for each participant. Only the research team will have access to the key to the identification code list. This list will be documented and secured by the Principal Investigator in accordance with GCP after completion of the study. No identification details will be reported in publications.

## Statistical methods

### Statistical methods for primary and secondary outcomes {20a} and subgroup analyses {20b}

Baseline characteristics and other descriptive data will be summarized using appropriate descriptive statistics. Continuous variables will be described using the mean and standard deviation, or the median and interquartile range, as appropriate based on the distribution. Categorical variables will be described using counts and percentages. Simpler parametric or non-parametric comparisons may be used for descriptive or supplementary analyses.

The main analytical framework for longitudinal primary and secondary outcomes will be regression-based repeated-measures analyses, primarily using linear mixed-effects models (LMEs) (and generalized mixed-effects models for non-continuous outcomes, where relevant). These models account for within-participant correlation over time and allow inclusion of participants with incomplete follow-up data under standard model assumptions. Analyses of intervention effects will include time, treatment group, and the time × treatment group interaction as key terms. Relevant covariates (e.g., age, sex, and baseline values), as appropriate for the specific outcome, will be included in adjusted analyses. Given the heterogeneity of disease severity in MCI and risk of baseline imbalance in a moderate sample size, if systematic baseline differences are observed across the groups, a multidimensional composite covariate may be developed and included as an adjustment variable.

Planned contrasts will include comparisons of the single-training group and double-training group versus the active-control group at 6 months as the main outcome. If the double-training group is significantly better than the active-control group at 6 months, the estimated mean difference between the double-training and single-training groups will be tested at the 5% significance level to assess a possible dose–response effect. Pairwise contrasts between groups will also be estimated at 3 and 12 months to assess immediate and longer-term effects. Multiple comparisons will be applied using Holm–Bonferroni across the relevant tests. Results will include estimated marginal means with 95% confidence intervals, mean difference between groups with 95% CIs, and standardized effect sizes (Cohen’s *d* derived using the pooled baseline standard deviation).

The primary analysis will include all participants who completed randomization (modified intention-to-treat approach). Sensitivity analysis may be performed as per-protocol analyses, analyses with adherence included as a covariate, or as multiple imputation or tipping point analysis for missing data, as appropriate.

Repeated MRI-derived outcomes will primarily be analyzed using LMEs. Cortical thickness and gyrification analyses will be conducted using FreeSurfer’s specialized longitudinal linear mixed-effects module (vertex-wise analyses), with adjustment for relevant covariates. To account for multiple comparisons in mass-univariate MRI analyses, results will be corrected using false discovery rate (FDR) methods (planned threshold *q* < 0.05). Other repeated MRI-derived measures (e.g., diffusion, perfusion, and QSM measures, where applicable) will be analyzed using longitudinal mixed-effects models.

Moderator analyses will be conducted to investigate characteristics associated with differential responses to working memory training. These analyses will use LME models with interaction terms (e.g., moderator × time × treatment, as appropriate) to evaluate whether intervention effects differ across demographic and biological factors. In addition, an exploratory Elastic Net regression approach will be used to identify baseline variables associated with high response, particularly in the presence of multiple correlated candidate predictors. Variables identified in exploratory models will be examined further in post-selection regression analyses and interpreted as hypothesis-generating.

Statistical analyses will be performed in Stata (version 18), except for vertex-wise cortical MRI analyses, which will be performed in FreeSurfer using its longitudinal LME module.

The economic analysis of mild cognitive impairment in Norway will be conducted by merging data from national health registries with detailed patient and relatives’ questionnaires.

### Methods in analysis to handle protocol non-adherence and any statistical methods to handle missing data {20c}

According to the methods described above, missing data will be reduced to a minimum. As for the primary outcome, imputations for missing data are not needed for a generalized mixed model. If missing data must be accounted for in the secondary outcomes, multiple imputation will be applied.

### Plans to give access to the full protocol, participant level-data, and statistical code {31c}

Restrictions imposed by the ethical committee prevent publicly sharing de-identified data. Data may be requested from the corresponding author according to research collaboration and data transfer guidelines. Availability is dependent on approval from The Norwegian regional committee for medical and health research ethics (REC).

## Oversight and monitoring

### Composition of the coordinating center and trial steering committee {5d}

The project-running group (Principal Investigator, postdoctoral fellow MRI, postdoctoral fellow glymphatics, postdoctoral fellow cognition, PhD candidates, and study secretary) oversees the project and meets monthly. Regularly, the wider team including the research nurses at the study centers meet 2–4 times a year. Both the advisory board and the User representative board meets with the Principal Investigator 2–3 times a year throughout the trial.

### Composition of the data monitoring committee, its role, and reporting structure {21a}

No DMC is established for the REACT MCI study based on the low-risk intervention in a non-critical population.

### Adverse event reporting and harms {22}

Computerized working memory-training programs might be tiresome but involve no harm to the participants. The craniospinal clearance investigation (glymphatics sub-study) is explorative in nature, and participants can opt out of inclusion to this sub-study. Moreover, there is a strict protocol in place regarding eligibility in this study arm. Although invasive in nature, the procedure is associated with minimal risk for participants. Any harm to participants will be registered in the trial database, and reported through the local institutions adverse events system.

### Plans for communicating important protocol amendments to relevant parties (e.g. trial participants, ethical committees) {25}

Changes to the protocol require the project-running group to seek permission from the Regional Ethics Committee.

### Dissemination plans {31a}

The results will be disseminated by scientific papers submitted to peer-review medical journals. Authorship will be determined based on the Vancouver criteria, and a written agreement regarding ownership of data and publications are signed by all project group members. The dissemination of study results will also be aimed at the general public by the www.reactmci.no website, social media such as Facebook and X, soft science publications and talks, and by using the networks within the patient and user organizations.

## Discussion

Currently, effective therapy for MCI is lacking. This multi-center randomized controlled trial assesses whether working memory training (20–25 sessions over 5 weeks) can improve cognitive function after 3, 6, 12, 24, and 48 months. Additionally, we will examine the microstructural effects of working memory training using MRI and explore the potential for identifying high responders by analyzing associations with glymphatic system function and genetic variations. Finally, we will evaluate the impact of working memory training on quality of life, caregiver stress, and its cost-effectiveness in individuals with MCI. Finding an intervention postponing the progression of MCI is of great importance for patients as well as for the global economy and health care. Moreover, identification of markers for training effects is important for future personalized interventions.

The 5-week working-memory training intervention leverages patients’ own resources, which is underexplored in MCI [28]. Most studies in the MCI-dementia continuum focus on disease modification or pathogenesis, with patient-centered outcomes like quality of life (QoL) and caregiver burden often underrepresented despite their importance to patients and caregivers [45]. Even small improvements or stabilization in health-related QoL (HRQoL) can be significant to the individual and thus provide cost-effective care.

To our knowledge, no studies have examined the effects of cognitive training interventions on caregivers. MCI impacts not only the affected individual but also caregivers, who report high levels of stress and, in some cases, depression or anxiety [46, 47]. The relationship between cognitive decline, neuropsychiatric symptoms, and caregiver burden is bidirectional [48]. Strengthening the ability of individuals to remain at home is crucial, especially in light of demographic changes.

Furthermore, this study gives insight in the socioeconomic impact of MCI in the Norwegian population, including hidden costs for the participants’ relatives. These data are currently unavailable in Norway.

This study challenges current practice in MCI by utilizing computer-based working memory training. Moreover, we aim to fill knowledge gaps regarding the length of training, the personalization of this intervention, as well as the effect on quality of life, caregiver stress, and its cost-effectiveness.

## Trial status

The trial started in May 2021. The current protocol is version 3.02 (dated 03/04/2024). Clinical trial identifier: NCT04792528. Last baseline assessment is expected in April 2025.

## Data Availability

Access to data will be governed by a written agreement between all participating centers and regulated by the Norwegian Regional Committee for Medical and Health Research Ethics (REK).
